# C1烟煤中自然产出的纳米二氧化硅对BEAS-2B细胞的体外毒性

**DOI:** 10.3779/j.issn.1009-3419.2012.10.01

**Published:** 2012-10-20

**Authors:** 光剑 李, 云超 黄, 拥军 刘, 律 郭, 永春 周, 堃 杨, 颖 陈, 光强 赵, 玉洁 雷

**Affiliations:** 1 650118 昆明，昆明医科大学第三附属医院/云南省肿瘤医院胸心外科 Department of Cardiothoracic Surgery, the Third Affiliated Hospital of Kunming Medical University/the Tumor Hospital of Yunnan Province, Kunming 650118, China; 2 650091 昆明，云南大学现代分析测试中心 Advanced Analysis and Measurement Center of Yunnan University, Kunming 650091, China; 3 650106 昆明，昆明贵研催化有限公司技术部 The Technical Department of Kunming Sino-Platinum Metals Catalyst Co. Ltd., Kunming 650106, China; 4 650118 昆明，昆明医科大学第三附属医院/云南省肿瘤医院肿瘤研究所 The Third Affiliated Hospital of Kunming Medical University/The Research Institute of Tumor Kunming 650118, China; 5 650032 昆明，昆明医科大学第一附属医院麻醉科 The department of Anesthesiology of the First Affiliated Hospital of Kunming Medical University, Kunming 650032, China

**Keywords:** C1烟煤, 纳米二氧化硅, 分离, 形貌特征, 体外毒性, C1 bituminous coal, Silica nanoparticle, Separation, Morphology characteristics, Vitro toxicity

## Abstract

**背景与目的:**

中国云南省宣威地区是世界非吸烟女性肺癌发病率最高的地区之一，其发病率是中国其他地方的20倍，前期研究认为，这种高肺癌发病率可能与当地出产和使用的烟煤燃烧产物中含有的二氧化硅颗粒物有关。本研究将从宣威地区出产的烟煤（C1烟煤）燃烧产物中分离二氧化硅颗粒物并进行表征，同时研究这种自然产出的二氧化硅颗粒物对正常人支气管上皮细胞（BEAS-2B）的体外毒性。

**方法:**

① 物理法从C1烟煤燃烧后的底灰中分离二氧化硅颗粒物，扫描电镜（scanning electron microscope, SEM）观察分离出的颗粒物形态，能谱分析显微组分的依存关系，透射电镜（transmission electron microscope, TEM）观察单颗粒形貌，激光粒度分析仪分析其颗粒物粒径分布，BET氮吸附比表面积仪测定颗粒物表面积；②应用3-(4, 5-dimethylthiazol-2-yl)-2, 5-diphenyltetrazolium bromide（MTT）比色法检测分离出的二氧化硅处理组（实验组）、工业生产的纳米二氧化硅处理组和结晶型二氧化硅处理组（对照组）的细胞成活率变化，测定经刺激24 h-72 h后细胞内活性氧化酶（ROS）和乳酸脱氢酶（LDH）含量变化。

**结果:**

① 我们从C1烟煤燃烧后的底灰中分离出二氧化硅颗粒物，这些颗粒物粒径大小不一，30 nm到120 nm的颗粒物占86.8%，形态各异，表面不光滑，赋存有铝、钙和铁等元素；②相同浓度下，与工业生产的纳米二氧化硅和结晶型二氧化硅相比，自然产出的纳米二氧化硅对BEAS-2B有更高的体外毒性。

**结论:**

① 物理法能从C1烟煤底灰中分离出天然的纳米二氧化硅颗粒物，且不改变原有的形貌特征和显微组分的依存关系；②天然产出的纳米二氧化硅因形貌不规则、高比表面积和复杂的化学组分可能比工业生产的纳米二氧化硅和结晶型二氧化硅具有更高的细胞毒性。

## Abstract (en)

**Background and objective:**

China's Xuan Wei County in Yunnan Province have the world's highest incidence of lung cancer in nonsmoking women-20 times higher than the rest of China. Previous studies showed, this high lung cancer incidence may be associated with the silica particles embedded in the production combustion from the C1 coal. The aim of this study is to separate the silica particles from production combustion from the C1 bituminous coal in Xuan Wei County of Yunnan Province, and study *in vitro* toxicity of naturally occurring silica particles on BEAS-2B.

**Methods:**

① Separating the silica particles from combustion products of C1 bituminous coal by physical method, observing the morphology by Scanning Electron Microscope, analysis elements by SEM-EDX, observed the single particle morphology by Transmission Electron Microscope, analyed its particle size distribution by Laser particle size analyzer, the surface area of silica particles were determined by BET nitrogen adsorption analysis; ②Cell viability of the experimental group (silica; naturally occurring), control group (silica; industrial produced and crystalline silica) was detected by assay used the 3-(4, 5-dimethylthiazol-2-yl)-2, 5-diphenyltetrazolium bromide (MTT) method, and the reactive oxygen species (ROS), lactate dehydrogenase (LDH) were determined after 24 h-72 h exposed to these particles.

**Results:**

① The physical method can separate silica particles from production combustion from the C1 bituminous coal, which have different size, and from 30 nm to 120 nm particles accounted for 86.8%, different morphology, irregular surface area and containing trace of aluminum, calcium and iron and other elements; ②Under the same concentration, the experiment group have higher toxicity on BEAS-2B than control groups.

**Conclusion:**

① Physical method can separate silica particles from production combustion from the C1 bituminous coal and not change the original morphology and containing trace; ②Naturally occurring silica nanoparticles have irregular morphology, surface area, and containing complex trace elements may has greater toxicity than the silica nanoparticle of industrial produced and crystalline silica.

中国云南省宣威地区（包括宣威、富源、麒麟和沾益等）（E 103°35′30″-104°49′48″, N 25°02′38″-26°44′50″），位于中国西南部，该地区拥有人口约310万，95%为农村居民，是世界非吸烟女性肺癌发病率最高的地区之一，甚至在宣威县的来宾镇，女性肺癌发病率高达400/100, 000，是全国平均水平的20倍^[1-3]^。

宣威地区女性肺癌高发病率受到国内外众多学者的关注，最近的研究^[4, 5]^表明，宣威地区女性肺癌高发可能与当地开采和使用的C1烟煤及其燃烧产物（底灰和烟尘）中含有的大量超细二氧化硅颗粒物紧密联系。1996年国际癌症研究机构（International Agency For Research On Cancer, IARC）将石英（结晶型二氧化硅）规定为人类第一类致癌物质，相关研究也支持石英是一种肺部致癌物质^[1]^，这些研究成果主要来自于对工业场所和职业环境暴露（如：喷砂、陶瓷工人、水泥制造、采石、建筑工人等）二氧化硅研究后获得。中国云南省宣威地区非吸烟女性肺癌可能是自然暴露（室内燃煤空气污染）二氧化硅后形成肺癌的良好模型。因此，从C1烟煤燃烧产物中分离天然产出的纳米二氧化硅颗粒物，分析这些颗粒物的粒径分布、表面特征和显微组分依存关系，研究这种自然产出的纳米二氧化硅是否比已知的有毒颗粒物（工业生产的纳米二氧化硅和结晶型二氧化硅）具有更高的体外细胞毒性，能为纳米二氧化硅致肺癌发生的假说提供理论支持，为纳米颗粒物毒性研究提供重要参考数据。

## 1 材料与方法

### 1.1 C1烟煤燃烧后底灰中的纳米二氧化硅分离与表征

#### 1.1.1 获取C1烟煤底灰样品

烟煤取自宣威县来宾镇老林煤矿和雁塘煤矿，当地人群肺癌发病率非常高，共20份，每份为1 kg。在可控气氛的管式电炉中进行灰化，把氮气和氧气预先混合（4:1的比例），将炉内温度加热至1, 100 oC，获得实验室烟煤燃烧后的高温灰化样品。

#### 1.1.2 C1烟煤底灰中的纳米二氧化硅分离

取约2 g底灰溶于8 mL的四氢呋喃溶液（Tetrahydrofuran, THF/杭州四季青公司）中，超声波浴缸中震荡（FS-60H, 130 W, 20 kHz, Fisher Scientific, Pittsburgh, PA, USA）约30 min，再加入六偏磷酸钠2 g（分散凝聚的细小颗粒），再次超声波震荡约10 min，静置2 h，待出现沉淀物后取上清液在高速离心机（H1650-W型/Xlang Yi有限公司)上离心（> 12, 000转，持续约10 min），去除上清液后自然风干，取沉淀物进行鉴定。

#### 1.1.3 二氧化硅颗粒物表征

扫描电镜（scanning electron microscopy, SEM）观察分离出的颗粒物形态及大小，用配带的能谱仪（Energy Dispersive X-ray analyzer, EDX）分析其颗粒物的显微组分；马尔文激光粒度分析仪分析颗粒物的粒径分布（将0.1 g的颗粒物溶于95%乙醇中，超声波浴缸中震荡分散颗粒物后上机分析。该仪器分析范围为0.02 μm-2, 000 μm，数据导出后在Excel表中得到分布曲线）；透射电子显微镜（transmission electron microscope, TEM）观察单颗粒形貌特征，配带的能谱仪分析单颗粒赋存的显微组分。

### 1.2 人正常支气管上皮细胞

BEAS-2B细胞株由昆明医科大学临床肿瘤学院肿瘤研究所提供，该细胞系主要用于筛选诱导或影响分化及致癌的化学或生物制剂。

### 1.3 主要化学试剂和仪器

四氢呋喃溶液、95%酒精、胎牛血清（杭州四季青生物公司），工业生产的50 nm二氧化硅、结晶型二氧化硅（Min-U-Sil 5）（U.S. Silica Company, Berkeley springs. Wv, USA）；色谱级正丁醇、乙腈、分析级1, 200万盐酸、胰蛋白酶（Pittsburgh, PA, USA）；Hank’s平衡盐溶液（Carlsbad, CA, USA）；磺酰罗丹明B，三氯乙酸（Irvine, CA, USA）；1, 1, 3, 3-四甲氧基丙烷、硫代巴比妥酸、2 -吡啶、2', 7'-二氯荧光素二脂（上海富众仪器有限公司）；L-丝氨酸、硼酸、二亚乙基三胺五乙酸（97%）（上海安谱科学仪器公司）；盐酸三氯三乙胺、丁基羟基甲苯、醋酸和磷酸二钠（北京华尔博科技公司）。QUANTA200型扫描电子显微镜（Philips Company, Holland），OXFORD型透视电子显微镜镜（Oxford, US），Phoenix+DIM一体化能谱及电子背散射衍射仪（EDAX Company, USA），Sartorius电子天平（Sartorius, Germany），高机能马弗炉FP310（YAMATO company, Japan），马尔文激光粒度分析仪（Malvern Instruments Ltd., USA），NOVA2000e型BET氮吸附比表面积仪（Quantachrome instruments Ltd/USA）。

### 1.4 细胞培养和二氧化硅处理

BEAS-2B细胞复苏后用含10%胎牛血清的Hams F-12（含L-谷氨酰胺）培养基中培养，培养基中加入100 U/mL的青霉素和100 μg/mL的链霉素，在37 ℃、5%CO_2_培养基中孵化。取对数生长期的BEAS-2B细胞接种于96孔培养板中，5×10^4^个细胞/孔。每组设3个复孔，待细胞贴壁48 h后加入刺激物悬浮液共同培养。共设3个处理组，宣威组（C烟煤中自然产出的二氧化硅）、50 nm工业生产的二氧化硅组和结晶型二氧化硅组（Min-U-Sil5），其基本特征见表 1。

**1:** 二氧化硅颗粒物表征 Characterization of the silica particles

	Size and distribution (nm) (Mean±SD)	Surface area (m^2^/g)	Metal impurity (dominant elements)
Silica (naturally occurring)	54±27	87.06	Ca, Fe, Al, Mn
Silica (industrially produced)	50±12	55.27	Na, Ca, Fe, Al
Silica (crystalline; Min-U-Sil5)	580±231	5.20	Ca, Fe, K, Al
The size, surface area were determined by Laser particle size analyzer and BET surface area analyzer, and the impurity mental elements by EDX.			

### 1.5 细胞成活率的测定

应用3-(4, 5-二甲基-2-噻唑)-2, 5-二苯基溴化四唑（methyl thiazolyl tetrazolium, MTT）比色法检测经处理后的BEAS-2B细胞成活率变化。

### 1.6 细胞毒性检验

细胞内活性氧化酶（reactive oxygen species, ROS）的测定：细胞内活性氧化酶通过2', 7'-二氯荧光素二乙酸酯方法测量（2', 7'-dichlorofluorescin diacetate, DCFH-DA），DCFH-DA进入细胞内和细胞内活性氧化酶结合形成荧光化合物二氯荧光素二乙酸酯。具体方法是：10 mM的DCFH-DA原液用Hank’s平衡盐溶液稀释500倍后作为工作液备用，待刺激物和BEAS-2B细胞共同培养24 h、48 h和72 h后用Hank’s平衡盐溶液洗涤2次，将洗涤后的细胞在2 mL的工作液中37 oC孵育30 min。荧光素通过酶标仪（Thermo Scientific Microplate Reader/USA）在485 nm和525 nm波长处测定。

乳酸脱氢酶（lactate dehydrogenase, LDH）水平测定：乳酸脱氢酶水平根据商业试剂盒操作指南进行测量（Pointe Scientific, Inc, Lincoln Park, MI, USA）。

### 1.7 统计学分析

用SPSS 17.0统计软件包进行统计学处理。每个实验重复3次，每次实验设3个复孔，所得数值采用Mean±SD表示，组间比较采用两样本*t*检验。*P* < 0.05为差异有统计学意义。

## 2 结果

### 2.1 C1烟煤底灰中分离出的纳米二氧化硅颗粒物与表征

C1烟煤燃烧后底灰中出现大量的游离微米级和纳米级二氧化硅颗粒物，呈纤维状或球形，赋存有钙、铁等元素（图 1）。底灰经反复多次分离后，可获得纯度较高的二氧化硅颗粒物，粒径分布多数为纳米级，少数为微米级，呈球形或不规则貌，与底灰中的二氧化硅颗粒物相比，其形貌未发生改变，分散较好，无明显团聚现象。能谱分析发现，二氧化硅颗粒物同时赋存少量的铝、钙、铁等元素（图 2），使用马尔文激光粒度分析仪进行颗粒物粒径分布分析，在可测量的范围内，发现30 nm-120 nm范围内的二氧化硅颗粒物占86.8%（图 3）。

**1:**
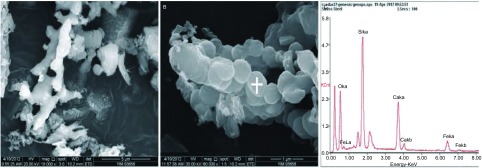
C1烟煤燃烧后底灰中的纤维状微米级二氧化硅颗粒物（A）和球形纳米二氧化硅颗粒物（B），能谱分析发现赋存有钙、铁等元素（C） The fibrous silica micronparticles (A) and spheral silica nanoparticles (B) in C1 coal ash, and containing traces of iron and calcium(C)

**2:**
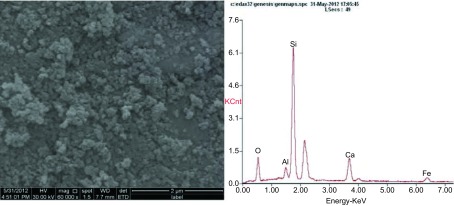
底灰中分离出的二氧化硅颗粒物形态及能谱分析，赋存钙、铁和铝元素 The picture of silica particles separated from coal ash and the EDX analysis, containing traces of iron, calcium and aluminum

**3:**
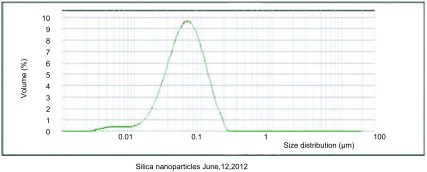
激光粒度分析结果，分离出的二氧化硅颗粒物主要分布在30 nm-120 nm之间 Particle size analysis by laser particle size analyzer, the silica particles are mainly distributed from 30 nm to 120 nm

通过透射电子显微镜进行单颗粒形态分析，发现这些颗粒物表面不光滑，形态不规整，少数呈纤维状，单颗粒主要成分为硅元素，赋存有铝、铁和锰等元素（图 4）。

**4:**
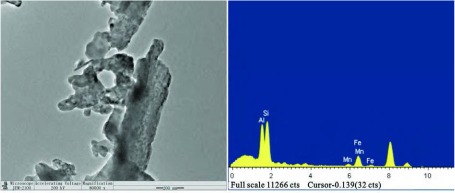
底灰中分离出的二氧化硅单颗粒形貌，表面不光滑，形貌不规则，赋存有铝、铁和锰元素 The morphology of single silica nanoparticles separated from coal ash, have irregular surface area and containing traces of iron, calcium and manganese

### 2.2 BEAS-2B细胞成活率变化

将0.1 g分离出的二氧化硅颗粒物溶于100 mL超纯水中，在超声波浴缸中震荡，配制成1 mg/mL的悬浮溶液为实验组（宣威组）。设相同浓度工业化生产的50 nm二氧化硅组、结晶型二氧化硅（Min-U-Sil 5）组和空白组为对照。待细胞贴壁48 h后，在培养基内分别加入配制的悬浮溶液，使刺激浓度分别达10 μg/mL、50 μg/mL和100 μg/mL。共同培养48 h后，应用MTT法检测细胞成活率变化情况。当刺激浓度为10 μg/mL时，与空白组相比，宣威组、50 nm二氧化硅组和结晶型二氧化硅组的细胞成活率分别为88.7%、89.1%和94.9%，宣威组的细胞成活率下降与结晶性二氧化硅组相比有差异（*P*=0.036, 7）；当二氧化硅刺激浓度为50 μg/mL时，其各组的细胞成活率分别为76.6%、87.6%和90.2%，宣威组的细胞成活率下降与50 nm二氧化硅组和结晶型二氧化硅组相比均有明显差异（*P*=0.007, *P*=0.001）；当刺激浓度为100 μg/mL时，各组的细胞成活率分别为57.3%、72.5%和78.7%，宣威组的细胞成活率下降与50 nm二氧化硅组和结晶型二氧化硅组相比均有明显差异（*P* < 0.05）（图 5）。虽然各组的细胞成活率均随二氧化硅刺激浓度的增加而下降，但以宣威组细胞成活率下降最为明显，其主要原因可能是在相同的质量下，从宣威烟煤燃烧底灰中提取的自然产出的二氧化硅颗粒物因形态不规则而具有较大的表面积，而颗粒物的表面积是影响其生物活性的最主要因素^[6-9]^。

**5:**
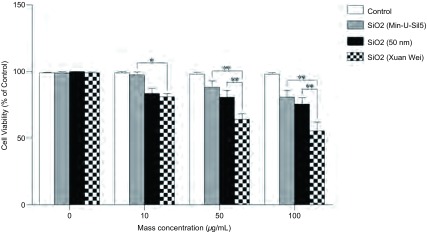
暴露10 *μ*g/mL、50 *μ*g/mL或100 *μ*g/mL自然产出的二氧化硅（宣威组）、工业生产的50 nm二氧化硅和结晶型二氧化硅48 h后BEAS-2B细胞成活率变化情况，数值采用均数±标准差表示。^*^表示细胞成活率下降组间比较有差异，^**^表示组间比较有明显差异 Changes in viability of BEAS-2B cells after 48 h exposure to 10 *μ*g/mL, 50 *μ*g/mL, or 100 *μ*g/mL of naturally-occurring silica nanoparticles (Xuan Wei group), 50 nm industrially-produced silica particles and crystalline silica (Min-U-Sil5). Values are the Mean±SD from three independent experiments. ^*^Indicates the decrease in cell viability was significantly different (*P* < 0.05), ^**^ (*P* < 0.01).

### 2.3 二氧化硅颗粒物对BEAS-2B细胞的体外毒性

待细胞贴壁48 h后，在培养基内分别加入配制好的悬浮溶液，使刺激浓度达100 μg/mL，待刺激培养基中的BEAS-2B细胞24 h、48 h和72 h后，测得各组BEAS-2B细胞的乳酸脱氢酶（LDH）水平逐渐升高，在相同时间点上，以宣威组细胞升高最为明显，与空白对照组相比分别升高了3.4%、9.2%和18.8%，结晶型二氧化硅组和50 nm二氧化硅组分别上升2.7%、3.8%、4.1%和5.2%、7.9%、10.3%。宣威组和结晶型二氧化硅组相比，在刺激48 h时，乳酸脱氢酶（LDH）升高有差异（*P*=0.027），在刺激72 h时有明显差异（*P*=0.001），与工业生产的50 nm二氧化硅组相比在各时间点均无明显差异。细胞培养基中的乳酸脱氢酶水平升高，提示存在细胞膜损伤，且上升越明显其细胞膜损伤越严重^[10]^。宣威烟煤燃烧后底灰中提取的自然产出的二氧化硅颗粒物因形态不规则，表面不光滑，部分呈纤维状而更容易穿透细胞膜而造成细胞膜损伤。因此，在相同的刺激时间、刺激浓度和相近的粒径下（50 nm组），宣威组的乳酸脱氢酶升高最明显（图 6）。

**6:**
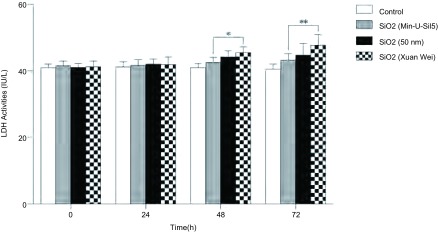
自然产出的纳米二氧化硅、结晶型二氧化硅和工业生产的纳米二氧化硅（50 nm）刺激24 h-72 h后，BEAS-2B细胞LDH水平变化情况，数值采用Mean±SD表示，^*^代表相同时间点上LDH水平上升组间比较有差异（*P* < 0.05），^**^代表LDH水平上升组间比较有明显差异（*P* < 0.01） The LDH level changes of BEAS-2B cells after 24 h-72 h exposure to naturally occurring silica nanoparticles (Xuan Wei group), 50 nm silica nanoparticles (industrial produced) and crystalline silica, values are Mean±SD from three independent experiments. ^*^Indicated the LDH level differentb (*P* < 0.05), ^**^ (*P* < 0.01)

细胞内活性氧化酶（reactive oxygen species, ROS）通过二氯荧光素（dichlorofluorescin, DCF）荧光强度来表示，DCF荧光强度随二氧化硅刺激时间的延长而增加。与空白组相比，当刺激时间为24 h时，宣威组的荧光强度增加了37.4%，与结晶型二氧化硅组增加的16.6%相比有差异（*P*=0.047, 2）；当刺激时间为48 h时，宣威组的荧光强度增加了48.1%，与结晶型二氧化硅组增加的26.8%相比有明显差异（*P* < 0.001），与50 nm二氧化硅组增加的30.1%相比有差异（*P*=0.031, 2）；当刺激时间为72 h时，宣威组的荧光强度增加了70.5%，与50 nm二氧化硅组增加的48.9%和结晶型二氧化硅增加的41.2%相比均有明显差异（*P* < 0.001, *P*=0.001）（图 7）。ROS是细胞代谢活化过程中产生的一系列活性氧簇，ROS的氧化能力强，广泛参与胞内信号的传递，能激活丝裂原活化蛋白激酶（mitogen-activated protein kinases, MAPKs）通路和转录因子复合物激活蛋白1（transcription factor complexes including activator protein-1, AP-1）通路。这些通路的激活可能促进炎症反应，增加感染，甚至与癌症发生相关^[11]^。

**7:**
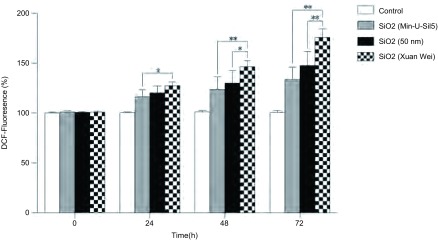
自然产出的纳米二氧化硅、结晶型二氧化硅和工业生产的纳米二氧化硅（50 nm）刺激24 h-72 h后，BEAS-2B细胞DCF荧光强度的变化。数值采用Mean±SD表示，^*^代表DCF荧光强度增加组间比较有差异（*P* < 0.05），^**^代表DCF荧光强度增加组间比较有明显差异（*P* < 0.01） The DCF-fluorescence increase of BEAS-2B cells after 24 h-72 h exposed to naturally occurring silica nanoparticles (Xuan Wei group), 50 nm silica nanoparticles (industrially produced) and crystalline silica. Values are Mean±SD from three independent experiments, ^*^indicated the DCF-fluorescence increase differently (*P* < 0.05), ^**^ (*P* < 0.01)

## 3 讨论

中国云南省宣威地区是我国非吸烟女性肺癌发病率最高的地区之一。研究表明，宣威地区肺癌高发与吸烟和工业污染等因素相关不明显，可能与当地开采和使用的C1烟煤中不同寻常的二氧化硅含量相关。这种假设主要是通过初步的煤地质化学评价，发现C1烟煤中相对富集的硅元素提出，但没有在统计学上定量，也没有观察二氧化硅颗粒物形态，然而，二氧化硅颗粒物形态在肺损伤方面是一个主要因素^[12]^。本研究的目的在于：①应用物理方法从宣威肺癌高发地区开采和使用的C1烟煤燃烧产物中分离出纳米二氧化硅，观察二氧化硅颗粒物的形态特征以及和其它元素之间的依存关系；②通过体外细胞实验，明确这种自然产出的纳米二氧化硅与工业生产的纳米二氧化硅和结晶型二氧化硅对正常人支气管上皮细胞的体外毒性有何不同。

### 3.1 C1烟煤燃烧底灰中的二氧化硅颗粒物分离与表征

我们应用物理方法从C1烟煤燃烧底灰中分离出的二氧化硅颗粒物，通过激光粒度分析后发现其粒经主要分布在30 nm到120 nm之间。颗粒物的粒径是重要的物理参数，它决定了颗粒物在人体呼吸系统中的沉积位置，粗颗粒物（2.5 μm-10 μm）主要附着在上呼吸道内，细颗粒（0.1 μm-2.5 μm）大部分沉积在下呼吸道中，而纳米颗粒物（< 0.1 μm）能深入末端支气管中，为颗粒物进一步迁移及毒害作用创造了条件^[13]^。我们从C1烟煤底灰中分离出的二氧化硅颗粒物多数为纳米级，这些颗粒物可以随着清理灰槽和抛洒底灰过程中产生的飞灰进入呼吸道。我们在调查中发现，宣威地区农村女性主要承担了清理灰槽和抛洒底灰作为农田肥料等工作，长时间吸入这些颗粒物可能对健康造成损害。另外，这种天然产出的纳米二氧化硅颗粒物形态各异，多成球形，少数呈纤维状，表面多不光滑，在相同质量浓度下，自然产出的纳米二氧化硅颗粒物比工业生产的纳米二氧化硅具有更大的表面积。能谱分析发现赋存有铝、钙和铁等元素，目前没有发现人类摄入钙和铁元素可能导致癌症发生的报道，但是，如果人类过多的摄入铝元素可能与癌症发生相关^[14]^。因此，从烟煤底灰中分离出的纳米二氧化硅颗粒物因其具有独特的粒径分布、巨大的比表面积和复杂的显微组分可能造成比工业生产的纳米二氧化硅和结晶型二氧化硅更严重的健康危害。

### 3.2 C1烟煤自然产出的纳米二氧化硅对支气管上皮细胞的体外毒性

物理方法从煤燃烧底灰中分离纳米二氧化硅颗粒物较化学方法简单、易行、廉价和分离过程无污染等特点，最重要的是这种方法没有改变原有纳米二氧化硅颗粒物的形貌、粒径和显微组份的依存关系。

通过使用从C1烟煤底灰中分离出的纳米二氧化硅颗粒物刺激体外培养的BEAS-2B细胞，发现对细胞成活率和细胞活性氧（ROS）水平的影响较明显，当刺激时间超过24 h，与工业化生产的纳米二氧化硅和结晶型二氧化硅相比均有差异，说明这种天然产出的纳米二氧化硅颗粒物可能具有比结晶型二氧化硅和工业生产的纳米二氧化硅更高的细胞毒性。这种自然产出的纳米二氧化硅颗粒物粒径大小不等、表面不光滑、形态不规则，巨大的表面积可保持很高的生物学活性，能导致氧化应激甚至DNA损伤，这些影响被认为可能与癌症发生相关^[15]^。另外，这种天然产出的纳米二氧化硅还赋存有铝、钙和铁等元素。因此，在进行C1烟煤中的纳米二氧化硅颗粒物细胞毒性研究方面，既要考虑这些颗粒物的粒径和形貌特征，还要考虑其显微组分的依存关系。

对于致癌物质的分离和鉴定，是一个非常复杂的工作，本研究的主要成果是用物理方法分离出了烟煤燃烧产物中的纳米二氧化硅颗粒物，保持了原有的理化特性，为进一步进行更深入的体外细胞实验和标准动物模型的建立提供了物质基础。
